# The neuronal protein Neurexin directly interacts with the Scribble–Pix complex to stimulate F-actin assembly for synaptic vesicle clustering

**DOI:** 10.1074/jbc.M117.794040

**Published:** 2017-07-14

**Authors:** Menglong Rui, Jinjun Qian, Lijuan Liu, Yihan Cai, Huihui Lv, Junhai Han, Zhengping Jia, Wei Xie

**Affiliations:** From ‡ Key Laboratory of Developmental Genes and Human Disease, Institute of Life Sciences, Southeast University, Nanjing 210096, China,; the § Institute of Life Sciences, the Collaborative Innovation Center for Brain Science, Southeast University, Nanjing 210096, China,; the ¶ Department of Neurosciences and Mental Health, The Hospital for Sick Children, Toronto, Ontario M5G 1X8, Canada, and; the ‖ Department of Physiology, Faculty of Medicine, University of Toronto, Toronto, Ontario M5S 1A8, Canada

**Keywords:** actin, cell adhesion, Drosophila, synapse, vesicles

## Abstract

Synaptic vesicles (SVs) form distinct pools at synaptic terminals, and this well-regulated separation is necessary for normal neurotransmission. However, how the SV cluster, in particular synaptic compartments, maintains normal neurotransmitter release remains a mystery. The presynaptic protein Neurexin (NRX) plays a significant role in synaptic architecture and function, and some evidence suggests that NRX is associated with neurological disorders, including autism spectrum disorders. However, the role of NRX in SV clustering is unclear. Here, using the neuromuscular junction at the 2–3 instar stages of *Drosophila* larvae as a model and biochemical imaging and electrophysiology techniques, we demonstrate that *Drosophila* NRX (DNRX) plays critical roles in regulating synaptic terminal clustering and release of SVs. We found that DNRX controls the terminal clustering and release of SVs by stimulating presynaptic F-actin. Furthermore, our results indicate that DNRX functions through the scaffold protein Scribble and the GEF protein DPix to activate the small GTPase Ras-related C3 Botulinum toxin substrate 1 (Rac1). We observed a direct interaction between the C-terminal PDZ-binding motif of DNRX and the PDZ domains of Scribble and that Scribble bridges DNRX to DPix, forming a DNRX–Scribble–DPix complex that activates Rac1 and subsequently stimulates presynaptic F-actin assembly and SV clustering. Taken together, our work provides important insights into the function of DNRX in regulating SV clustering, which could help inform further research into pathological *neurexin*-mediated mechanisms in neurological disorders such as autism.

## Introduction

Neurons are the basic unit of the nervous system, and they communicate with each other through synapses. After being captured by our sensory organs, neural signals pass between synapses in the form of neurotransmitters. As the vehicles of neurotransmitters, synaptic vesicles (SV)^2^ are essential for neurotransmission. SVs can be divided into three distinct pools according to their localization and function. The SVs adjacent to the active zone and ready to be released are referred to as the ready release pool. The second pool of vesicles is the exo/endo-cycling pool, which is also found close to release sites and supplies the ready release pool. Finally, the pool located away from the active zone, and that contains the majority of SVs, is referred to as the reserve pool and is considered to be a storage pool (1, 2). Actin is a major part of the cytoskeleton that is required for maintaining the architecture of synapses as well as contributing to their function (3, 4), and a significant amount of evidence has shown that the localization, translocation, and release of SVs can be altered by disturbing the polymerization of pre-synaptic actin (5, 6).

The synapse is a highly specialized structure, and synaptogenesis is a highly complex process. Previous studies have shown that synaptic adhesive molecules play important roles in synaptogenesis and neurotransmission (7), and a number of synaptic cell adhesion molecules, including Neuroligins and Neurexins have been identified over the past few decades. Neurexin was first recognized as a receptor for α-latrotoxin, a black widow spider venom component that triggers massive neurotransmitter release (8). There are three *neurexin* genes in mammals, each of which has two promoters generating α-Neurexin and β-Neurexins, whereas there is only one *neurexin-1* gene in *Drosophila* (*dnrx*) (9). Recent studies both in *Drosophila* and mammals showed that Neurexin plays a significant role in synaptic architecture and function, and there is evidence suggesting that *neurexin* is associated with autism spectrum disorders (ASDs) (10, 11). The complexity and redundancy of the *neurexin* genes in mammals motivated us to focus on simpler model systems, such as *Drosophila*, to investigate the *in vivo* function of DNRX.

Neurexin has been shown to bind to several molecules, including the presynaptic scaffolding proteins Mint (12), CASK (13, 14), and LRRTM2 (15, 16). Recently, DNRX also has been demonstrated to interact with the *N*-ethylmaleimide sensitive factor to regulate short-term synaptic depression and to interact with Spinophilin to maintain active zone architecture (17, 18). A typical trans-synaptic complex is formed by the heterophilic interaction of presynaptic Neurexins and postsynaptic Neuroligins (19, 20), and these complexes have attracted much attention as scaffolding complexes that not only maintain the normal structure of the synapse but also function in passing signals across the synapses. It is thus clear that Neurexin is a multifunctional molecule. Despite the identification and characterization of these proteins that functionally associated with DNRX, our understanding of the pathways including DNRX that control synaptic function are still incomplete, with other partners and mechanisms yet to be uncovered and analyzed.

In this study, we have investigated the role of DNRX in the cluster and release of SVs at synaptic terminals. We demonstrate that the effect of DNRX is mediated by presynaptic F-actin and there is a direct interaction between the C-terminal PDZ-binding motif of DNRX and the PDZ domains of the tumor suppressor protein Scribble. Furthermore, we find that Scribble bridges DNRX to DPix, forming a DNRX–Scribble–DPix complex to activate Rac1 and affect presynaptic F-actin assembly and SV clustering. Taken together, these studies provide novel insight into the mechanisms underlying the regulation of neurotransmitter release by DNRX.

## Results

### The clustering and release of SVs at the synaptic terminal is altered in dnrx mutants

To better understand the role of Neurexin at the synapse, we investigated whether there are some additional defects after ablating *dnrx*. Previous studies both in mammals and *Drosophila* have shown that Neurexin affects neurotransmitter release, and that the excitatory junction potential (EJP) amplitude and miniature EJP (mEJP) frequency are altered in the *neurexin* mutant (21, 22). Therefore, we examined whether the localization and membrane fusion of SVs would be affected in a *dnrx* mutant. As a marker of synaptic activity, we used Synaptotagmin (SYT), which is a synaptic vesicle membrane protein and calcium sensor that is efficiently transported to presynaptic terminals and is expressed in specific regions of the synapse (23), to indicate SV.

**Figure 1.**
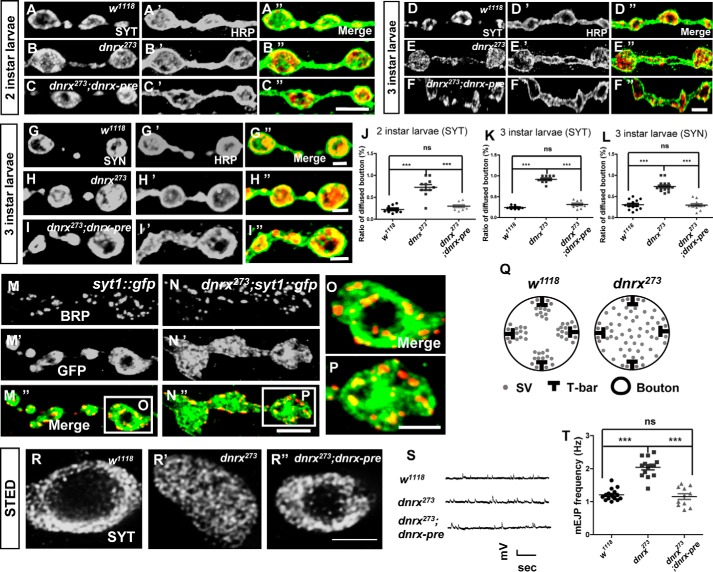
**DNRX is necessary for synaptic terminal aggregation and release of SVs.** *A–C*″, synaptic boutons of *wild-type* (*A–A*″), *dnrx* mutant (*B–B*″), and pre-synaptic rescue (*C–C*″) at two instar larvae stage double stained for SYT (*red*) and HRP (*green*), which labels the pre-synaptic SVs and neuronal membrane, respectively. *D–F*″, synaptic boutons of *wild-type* (*D–D*″), *dnrx* mutant (*E–E*″), and pre-synaptic rescue (*F–F*″) at third instar larvae stage double stained for SYT (*red*) and HRP (*green*), which labels the pre-synaptic SVs and neuronal membrane, respectively. *G–I*″, synaptic boutons of *wild-type* (*G–G*″), *dnrx* mutant (*H–H*″), and pre-synaptic rescue (*I–I*″) at third instar larvae stage double stained for SYN (*red*) and HRP (*green*), which labels the pre-synaptic SVs and neuronal membrane, respectively. *J,* quantification of the SYT-diffused boutons ratio at two instar larvae stage in muscle 4 shows that *dnrx* mutants have disturbed the distribution of SVs and this phenotype can be rescued by pre-synaptic DNRX. *K,* quantification of the SYT-diffused boutons ratio at the third instar larvae stage in muscle 4 shows that *dnrx* mutants have disturbed the distribution of SVs and this phenotype can be rescued by pre-synaptic DNRX. *L,* quantification of the SYN-diffused boutons ratio at third instar larvae stage in muscle 4 shows that *dnrx* mutants have disturbed the distribution of SVs and this phenotype can be rescued by pre-synaptic DNRX. *M–N*″, confocal images of third instar larvae NMJ boutons double labeled with anti-BRP (*red*) and anti-GFP (*green*) in *syt::gfp* (*M–M*″), and *dnrx^273^,syt::gfp* mutants (*N–N*″) showing that loss of DNRX disrupts the distribution of terminal SVs. *O–P,* amplified confocal images of single third instar larvae NMJ bouton double labeled with anti-Brp (*red*) and anti-GFP (*green*) in *syt::gfp* (*O*), and *dnrx^273^,syt::gfp* (*P*) showing the dispersed distribution of SVs in *dnrx* mutants. *Q,* representative diagrammatic sketch of SVs in single bouton of *wild-type* and *dnrx* mutant. *R–R*″, STED images of third instar larvae single bouton of *wild-type* (*R*), *dnrx* mutant (*R′*), and pre-synaptic rescue (*R*″) labeled with SYT, showing the SVs were diffused in *dnrx* mutant and can be rescued by pre-synaptic DNRX. *S*, representative traces of spontaneous responses of indicated genotypes. *T,* quantification of mEJP frequency of the indicated genotypes, showing that the mEJP frequency was increased in *dnrx* mutant and the DNRX could fully rescue the defects at pre-synapse. Data are mean ± S.E. ***, *p* < 0.001; **, *p* < 0.01; and *, *p* < 0.05. *ns*, not significant. Two-tailed Student's *t* tests were used to compare genotypes. *Scale bar*, 5 (*A–C*″), 5 (*D–F*″), 2 (*G–G*″), 2 (*H–H*″), 2 (*I–I*″), 5 (*M–N*″), 2.5 (*O–P*), and 2.5 μm (*R–R*″).

To assess the defects of SVs in *dnrx* mutants, we observed the synaptic terminal SV distribution of *Drosophila melanogaster* neuromuscular junction (NMJ) at different development stages of larvae from 2 to 3 instar. We found that loss of DNRX resulted in an ∼200% increase in the ratio of SYT-diffused boutons when compared with controls. Just as we expected, this defect could be fully rescued by overexpressing full-length *dnrx* cDNA within the pre-synapse in the *dnrx* mutant at 2 instar larvae (Fig. 1, *A–C*″ and *J*). When it turned to 3 instar larvae we got the same result for losing of DNRX resulted in an ∼300% increase in the ratio of SYT-diffused boutons when compared with controls (Fig. 1, *D–F*″ and *K*). Synapsin (SYN) is also a SV protein, which is localized at the SV membrane and can be used as another SV marker. We observed that the distribution of synaptic terminal SVs is diffused in *dnrx* mutant (Fig. 1, *G–I*″ and *L*). To be able to show the relative position of active zone (AZ) and SVs at the same time, we employed the *syt::gfp* knock-in fly with an integrated *dnrx* mutant background. Confocal images of third instar larvae NMJ boutons double labeled with active zone protein Bruchpilot (BRP) and GFP showed that loss of DNRX disrupted the distribution of terminal SVs (Fig. 1, M–*P*). The representative diagrammatic sketch illustrates the distribution of SVs in the *dnrx* mutant (Fig. 1*Q*). To make our measurements more precise, we turned to stimulated emission depletion (STED) microscopy to visualize the terminal distribution of SVs, and we observed the same phenotype (Fig. 1, *R–R*″). To determine whether the number of SVs and synapses were changed in *dnrx* mutants, we detected the number and density of AZ in *wild-type* and *dnrx* mutant and the immunostaining results showed that the number of AZ of single bouton is increased, whereas the AZ density of single bouton is decreased in *dnrx* mutant (supplemental Fig. S1, *A–D*). Moreover, we analyzed the total protein levels of SYN and BRP in adult heads with antibodies, and there were no differences between *dnrx* mutants and *wild-type* flies (supplemental Fig. S2, *A–C*).

The defect of SV localization was reminiscent of SV release. Thus, we also explored whether the release of SVs was altered in *dnrx* mutants. We observed profound abnormalities in the spontaneous release of SVs in the *dnrx* mutant and found that it can be restored to a normal level after inducing full-length DNRX within the pre-synapse in the *dnrx* mutant (Fig. 1, *S–T*). Collectively, these results suggest that DNRX is essential for the proper aggregation and release of SVs at synaptic terminals.

### The effects of DNRX on SV aggregation and release depend on presynaptic F-actin

Studies in mammals have suggested that cell adhesion complexes can localize presynaptic SVs by regulating local actin polymerization (24). Actin is one of the most prominent cytoskeletal molecules at synapses, and it is especially abundant at presynaptic terminals and postsynaptic dendritic spines (25). Moreover, pharmacological studies revealed a role for subsynaptic distribution of F-actin in SV mobilization and exocytosis (26). Studies in mammals have reported that treatment with latrunculin-A (an actin monomer sequestering agent) completely disrupts the actin arrangement and interferes with SV dynamics (4, 26). Conspicuously, actin participates in a regulatory mechanism at the synaptic terminals that inhibits the fusion of SVs at the active zone (26, 27). These observations indicate that DNRX might affect the distribution and release of SVs by regulating the presynaptic actin arrangement.

**Figure 2.**
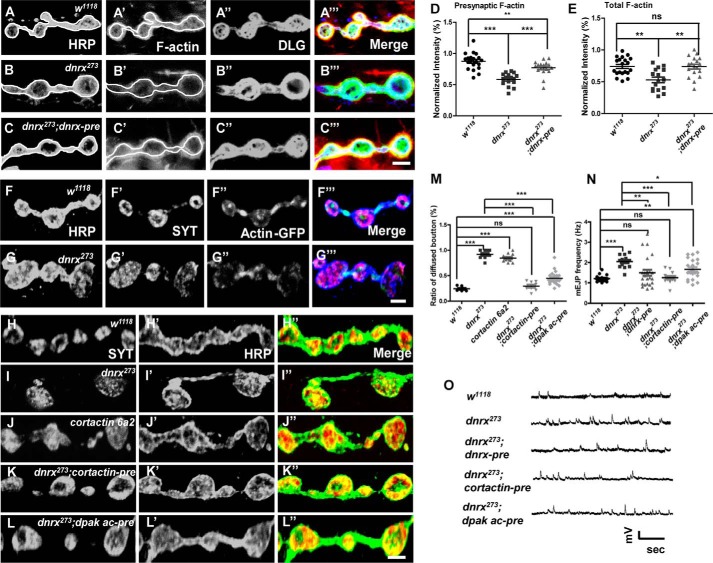
**DNRX regulates SV clustering and release dependent on presynaptic F-actin.** *A–C*‴, confocal images of third instar larvae NMJ type Ib boutons at muscles 12/13 triple labeled with Texas Red phalloidin (*red*), anti-DLG (*green*), and anti-HRP (*blue*) in *wild-type* (*A*), *dnrx* mutants (*B*), *ok6*>*dnrx* rescue (*C*). The phalloidin signal contained in *white circles* correspond to HRP, largely reflecting the presynaptic F-actin. *D* and *E,* summary graph of relative fluorescence intensity of F-actin correspond to HRP (*D*) and DLG (*E*) showing that the F-actin fluorescence intensities were significantly reduced in *dnrx* mutant and can be restored to normal levels. *F–G*‴, representative confocal images of third instar larvae NMJ labeled with anti-HRP (*blue*), anti-SYT (*red*), and anti-GFP (*green*) in *wild-type* and *dnrx* mutant, respectively. The *light green spot* represents the pre-synaptic F-actin decreased in the *dnrx* mutant. *H–L*″, synaptic boutons of *wild-type* (*H–H*″), *dnrx* mutant (*I–I*″), *cortactin* mutant (*J–J*″), and pre-synaptic overexpress Cortactin (*K–K*″), and the active form of DPak (*L–L*″) in the *dnrx* mutant double stained for SYT (*red*) and HRP (*green*), which labels pre-synaptic SVs and neuronal membrane, respectively. *M,* quantification of SYT-diffused bouton ratio shows presynaptic branched F-actin can fully rescue the diffused distribution of SVs in *dnrx* mutant. *N,* quantification of mEJP frequency of the indicated genotypes, showing that mEJP frequency was increased in *dnrx* mutant and the Cortactin, the active form of DPak could rescue the defects at pre-synapse. *O,* representative traces of spontaneous responses of the indicated genotypes. Data are mean ± S.E. ***, *p* < 0.001; **, *p* < 0.01; and *, *p* < 0.05. *ns*, not significant. Two-tailed Student's *t* tests were used to compare genotypes. *Scale bar*, 5 (*A–C*‴), 5 (*F–G*‴), and 5 μm (*H–L*″).

To determine whether the impact of DNRX on SV localization depends on presynaptic F-actin, we used Texas Red-conjugated phalloidin and fluorescently tagged actin (GFP-actin) to determine the distribution and amount of F-actin. We found that the fluorescence intensity of F-actin was significantly reduced in *dnrx* mutants and that this defect could be restored to normal levels after introducing full-length DNRX at the pre-synapse (Fig. 2, *A–C*‴). Furthermore, we measured the fluorescence intensity of F-actin in relationship to neuronal plasma membrane (HRP) and Discs large (DLG), which can be used to reflect the pre-synaptic and total F-actin, respectively. We found that both pre-synaptic and total F-actin were decreased in the *dnrx* mutant and could be rescued by introducing full-length DNRX (Fig. 2, *D* and *E*). GFP-tagged actin has previously been used to observe the localization of actin at synaptic terminals, and previous research has shown that these bright spots most likely correspond to F-actin (28). When we focus on the distribution of presynaptic F-actin, GFP-tagged actin has been confirmed to be particularly useful. We found that the presynaptic bright spots decreased in the *dnrx* mutant compared with *wild-type* (Fig. 2, *F* and *G*‴), suggesting that DNRX positively regulates F-actin recruitment to the synapse.

To investigate the role of pre-synaptic F-actin in DNRX-regulated clustering of SVs at synaptic terminals, we adjusted the polymerization of actin at the pre-synapse by controlling the expression of Cortactin and the active form of DPak (*Drosophila* Pak). Cortactin is an actin-associated protein that promotes the polymerization of actin by stabilizing Arp2/3 complexes (29), whereas Pak regulates G-actin nucleation through Limk/Coffilin signaling (30). Our results suggested that the defects in SV clustering at presynaptic terminals were rescued by expressing Cortactin and the activated form of DPak in neurons using motor neuron-specific Gal4 (OK6-Gal4) (Fig. 2, *H–L*″ and *M*), although the rescue extent was different. It may involve distinct F-actin-regulating forms by these two proteins.

To further test the mechanism through which DNRX modulates the spontaneous release of SVs, we attempted to rescue the abnormality in the spontaneous release of SVs in the *dnrx* mutant. The result showed that Cortactin and activated DPak could rescue the abnormality (Fig. 2, *N–O*). In addition, we also measured the protein level of Cortactin and Arp2 in the *dnrx* mutant, and we saw no effect of DNRX on the amount of Arp2 or Cortactin (supplemental Fig. S2, *A*, *D*, and *E*). Taken together, these results suggest that the effect of DNRX on SV aggregation and release depends on presynaptic F-actin.

### DNRX is co-localized with Scribble in the nervous system

Scribble is a cytoplasmic multimodular protein, and a previous study showed that mammalian Scribble regulates epithelial cell adhesion and migration (31). The *Drosophila* homologue of Scribble is located at the basolateral membrane and the septate junctions of epithelia (32). Recently, Scribble has been found to play essential roles in the nervous system (33, 34) and in active forgetting (35), a process in which *dnrx* was found to be involved (36). Loss of function of Scribble causes numerous defects in the nervous system, including alterations in synaptic architecture and function. DNRX is abundantly expressed in neurons in the central nervous system and plays important roles in associative learning in *Drosophila* (9, 20), and DNRX can also be detected, and it functions in the *Drosophila* NMJ (21, 37, 38). Thus we hypothesized that DNRX functions together with Scribble.

**Figure 3.**
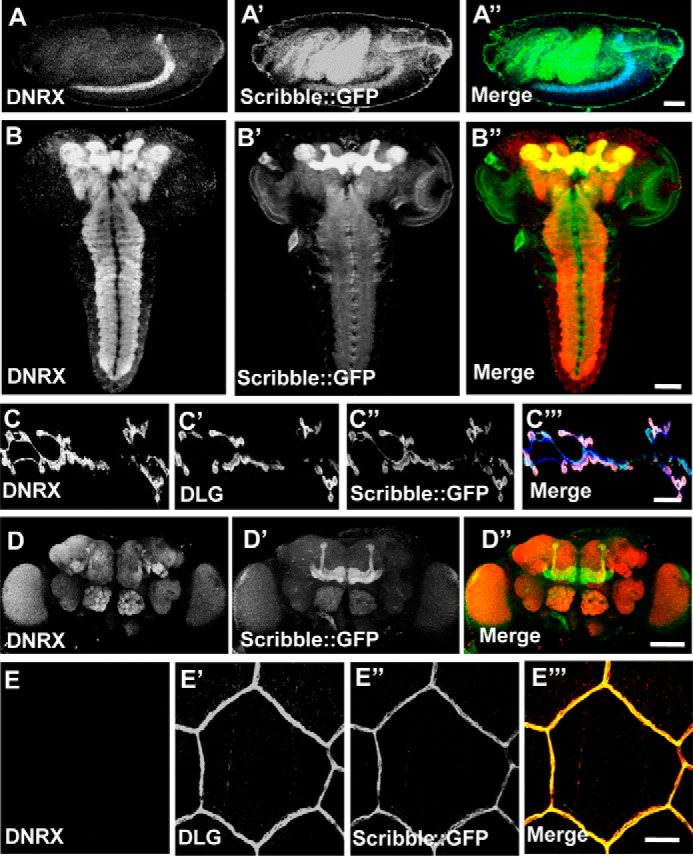
**DNRX is co-localized with Scribble in the nervous system.** *A–A*″, staining for *scribble::gfp* knock-in fly embryo with anti-DNRX (*blue*) and GFP is the spontaneous green fluorescence, showing that DNRX and Scribble are co-localized in the central nervous system. *B–B*″, third instar larvae brains of *scribble::gfp* staining with anti-DNRX (*red*) and GFP is the spontaneous green fluorescence, showing that DNRX and Scribble are co-localized in the central nervous system especially concentrated at mushroom body. *C–C*‴, confocal images of the third instar larvae neuromuscular junction at muscles 6/7 staining with anti-DNRX (*blue*), anti-DLG (*red*), and GFP is the spontaneous green fluorescence in *scribble::gfp* integrated with the DNRX overexpression fly, showing that DNRX and Scribble were co-localized in neuromuscular junction. *D–D*″, *scribble::gfp* knock-in fly adult brain staining with anti-DNRX (*red*) and GFP are the spontaneous green fluorescence, showing that DNRX and Scribble are co-localized in the central nervous system. *E–E*‴, epithelial cell staining with anti-DNRX, anti-DLG (*red*), and GFP is the spontaneous green fluorescence, showing that DNRX and Scribble are not co-localized at epithelial cells. *Scale bar*, 50 (*A–A*″), 50 (*B–B*″), 20 (*C–C*‴), 100 (*D–D*″), and 20 μm (*E–E*‴).

We used a GFP knock-in fly to mark endogenous Scribble, and to determine whether DNRX and Scribble are co-localized in the nervous system. At the embryonic stage, the immunostaining showed that DNRX was highly co-localized with Scribble in the developing nervous system (Fig. 3, *A–A*″). We also found that DNRX and Scribble were co-localized in the central nervous system at the third instar larval stage, and they were abundantly expressed and highly co-localized in the mushroom body. Moreover, they were co-localized in the ventral nerve cord with different expression levels (Fig. 3, *B–B*″). Previously, we found that DNRX could also be detected in a small subset of peripheral nervous system neurons (9, 38). Because of the low sensitivity of the DNRX antibody, we used motor neuron-specific Gal4 (OK6-Gal4) to drive DNRX expression and found that DNRX and Scribble were co-localized at NMJs of *Drosophila* (Fig. 3, *C–C*‴). Finally, we also observed a similar distribution of DNRX and Scribble in the heads of adult *Drosophila*, such as antennal lobe and mushroom body with different expression levels (Fig. 3, *D–D*″). However, in epithelial cells, we could only detect the signal from Scribble (Fig. 3, *E–E*‴). Collectively, these results show that DNRX and Scribble are specifically co-localized in the nervous system at all developmental stages of *Drosophila*.

### DNRX physically interacts with Scribble PDZ domains through C-terminal PDZ-binding motif

**Figure 4.**
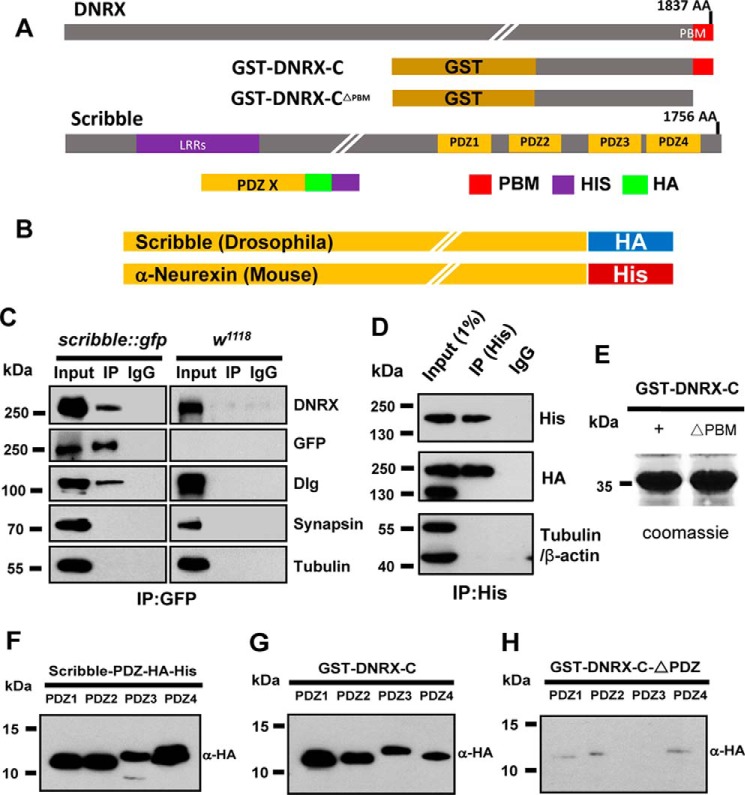
**DNRX binds with the PDZ domains of Scribble via the C-terminal PDZ-binding motif.** *A,* schematic representation of the protein structure of DNRX and Scribble. The polypeptide structure of the GST-fused DNRX C-terminal section with and without the C-terminal PDZ-binding motif and the PDZ domains of Scribble with HA and HIS tags. *B,* schematic graph of full-length HA-tagged Scribble of *Drosophila* and HIS-tagged α-Neurexin of mouse constructs. *C,* immunoprecipitated (*IP*) from whole *Drosophila* adult brain extracts with GFP nano-antibody fused beads in *scribble::gfp* and *wild-type* strains, respectively. Anti-GFP antibody precipitated the DNRX from the lysates, whereas the control IgG and *wild-type* sample did not. Suggesting DNRX physically interacts with Scribble *in vivo*. Input is 1%. *D,* Western blots of anti-His immunoprecipitates using protein lysate of HEK293 cells that can express the proteins of HA-Scribble and HIS-α-Neurexin, showing that HA-Scribble was coimmunoprecipitated with HIS-α-Neurexin in mouse. *E,* Coomassie Brilliant Blue for the purified DNRX C-terminal protein and deleted final 7-amino acid protein. *F,* Western blot validation for the purified 4 PDZ domain polypeptides of Scribble. *G* and *H,* GST pulldown with the DNRX C-terminal protein and deleted a very C-terminal 7-amino acid protein, respectively. Incubating with 4 PDZ domains of Scribble. Western blot results showed DNRX directly binds with all the PDZ domains of Scribble.

Previous studies have shown that Scribble belongs to the LAP protein family and consists of 16 leucine-rich repeats and 4 PDZ domains (Fig. 4*A*). The 4 PDZ domains have been shown to interact with several molecules that function both in neurons and epithelial cells. In mammals, it has been shown that Scribble can interact with the C-terminal PDZ-binding site of β-catenin to localize SVs to synapses through the PDZ domain (39). In addition, it has been shown that Scribble can form a complex with βPix to regulate actin arrangements and SV assembly (40). Recently, it has been found that NOS1AP associates with Scribble and regulates dendritic spine development (41).

To test whether there is a physical interaction between DNRX and Scribble, we used lysates from adult heads for co-immunoprecipitation assay, and found that anti-GFP antibody was able to immunoprecipitate DNRX (Fig. 4*C*). This provided evidence that DNRX can form a complex with Scribble *in vivo*. To validate a conserved interaction between Scribble and Neurexin, we cotransfected murine α-Neurexin with a HIS tag and *Drosophila* Scribble with an HA tag (Fig. 4*B*) into HEK293 cells. After inducing and extracting these two proteins we incubated them together. Co-immunoprecipitation experiments showed that *Drosophila* Scribble physically interacts with murine α-Neurexin (Fig. 4*D*). Therefore, the physical interaction between Neurexin and Scribble in the central nervous system is conserved at least between *Drosophila* and mouse.

To further confirm the interaction between DNRX and Scribble, we performed a GST pulldown assay. We constructed the C-terminal portion of DNRX with or without the PDZ-binding motif (Fig. 4*E*), and we generated four polypeptides of each Scribble PDZ domain with HA and HIS tags (Fig. 4*F*). Our GST pulldown results showed that the full C-terminal portion of DNRX was able to efficiently pulldown each of the four Scribble PDZ domains (Fig. 4*G*). However, deletion of the very C-terminal seven amino acids containing the PDZ-binding motif of DNRX significantly reduced the affinity between DNRX and Scribble (Fig. 4*H*). These results strongly support the idea that the very C-terminal PDZ-binding motif of DNRX directly binds to the Scribble PDZ domains *in vitro*.

### The interaction between DNRX and Scribble is required for the aggregation of SVs

**Figure 5.**
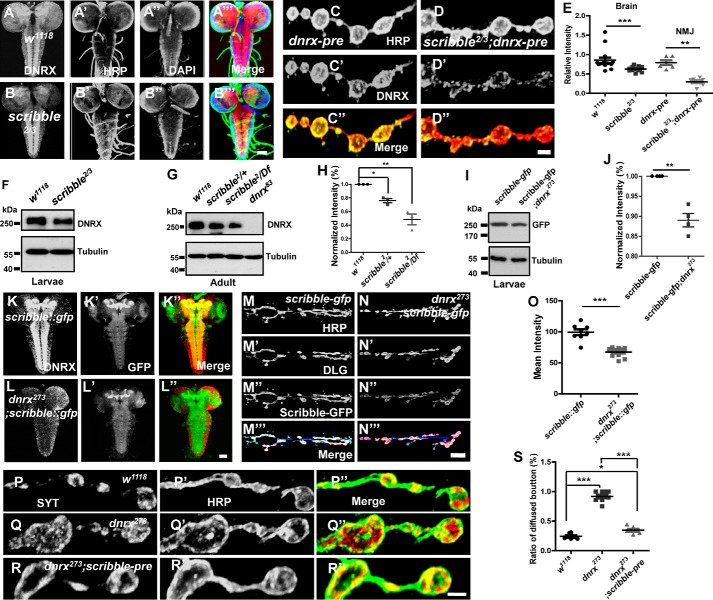
**DNRX interacts with Scribble and sustains the terminal SV aggregation.** *A–B*‴, representative images of larvae brains of the indicated genotypes were stained with DNRX (*red*), HRP (*green*), and DAPI (*blue*), showing the level of DNRX was decreased in *scribble* mutant. *C–D*″, representative images of larvae NMJ of the indicated genotypes were stained with DNRX (*green*) and HRP (*red*), showing the level of DNRX was decreased in the *scribble* mutant. *E,* quantification of the fluorescence intensity for DNRX both in brain and NMJ of the indicated genotypes. *F* and *G,* Western blot analysis of protein lysates prepared from heads using anti-DNRX antibody showing that the level of DNRX was reduced in *scribble* mutant both in larvae (*F*) and adult (*G*) stages. *H,* quantitative analysis of data for Western blot analysis of the indicated genotypes for adult flies. *I,* Western blot analysis of protein lysates prepared from heads using anti-GFP antibody showing that the level of Scribble was reduced in the *dnrx* mutant in larvae stage. *J,* quantitative analysis for Western blots of the indicated genotypes. *K–L*″, representative images of larvae brain of the indicated genotypes stained with DNRX (*red*) and GFP (*green*), showing the level of Scribble was decreased in *dnrx* mutant. *M–N*‴, representative images of larvae NMJ of the indicated genotypes were stained with HRP (*blue*), DLG (*red*), and GFP (*green*), showing the level of Scribble was decreased in *dnrx* mutant. *O,* quantification of the fluorescence intensity for Scribble::GFP of the indicated genotypes. *P–R*″, synaptic boutons of *wild-type* (*P–P*″), *dnrx* mutant (*Q–Q*″), and pre-synaptic overexpress Scribble in *dnrx* mutant (*R–R*″) double stained for SYT (*red*) and HRP (*green*), which labels the pre-synaptic SVs and neuronal membrane, respectively. *S,* quantification of the ratio of SYT-diffused boutons in muscle 4 shows that *dnrx* mutants have disturbed the terminal cluster of SVs and this phenotype can be rescued by pre-synaptic Scribble. Data are mean ± S.E. ***, *p* < 0.001; **, *p* < 0.01; and *, *p* < 0.05. Two-tailed Student's *t* tests were used to compare genotypes. *Scale bar*, 50 (*A–B*‴), 5 (*C–D*″), 50 (*K–L*″), 20 (*M–N*‴), and 5 μm (*P–R*″).

Because the data above showed that DNRX could directly bind to Scribble through the PDZ-binding motif, we hypothesized that a functional relationship exists between these two proteins. To test this, we measured the expression pattern of DNRX in *scribble*-null mutants. Surprisingly, the immunostaining results both in the brain and NMJ showed that the level of DNRX was significantly reduced compared with controls (Fig. 5, *A–D*″ and *E*), and Western blots confirmed that the protein level of DNRX was clearly decreased in *scribble* mutants compared with *wild-type* (Fig. 5, *F–H*) in a dose-dependent manner.

We also sought to determine whether DNRX has an impact on the level and localization of Scribble at the larval NMJ. Because there are no antibodies available against Scribble, and Scribble is expressed at both the pre- and post-synapse (33, 41), we used the GFP knock-in fly to reflect the endogenous Scribble. We observed that the level of GFP fused Scribble in the *dnrx* mutant was decreased both in the central nervous system and NMJ when compared with controls (Fig. 5, *I–O*). We performed qRT-PCR to determine whether DNRX affects the transcription of Scribble, but we found no statistical difference in the mRNA level of Scribble between *wild-type* and *dnrx* mutant (supplemental Fig. S3*A*). Thus DNRX regulates Scribble mostly at the protein level and it is possible that DNRX and Scribble are stabilized by each other. Together, these results suggest that DNRX regulates the recruitment of Scribble to the synapse and that the levels of DNRX and Scribble in the synapse are stabilized by each other.

To better understand the effect of Scribble on SV distribution we assessed the terminal SV in *scribble* mutant and we found that SVs are diffused in bouton after ablating Scribble (supplemental Fig. S3, *B–C*″). To further determine the relationship between DNRX and Scribble, we next investigated the functional significance of Scribble in DNRX-dependent aggregation of SVs. We independently overexpressed Scribble in the *dnrx* mutant background and the immunostaining results showed that the ratio of SYT-diffused boutons was decreased when compared with the *dnrx* mutant and was nearly restored to *wild-type* level (Fig. 5, *P–R*″ and *S*). In summary, these results suggest that DNRX interacts with Scribble and that this complex is required for sustaining normal SV clustering.

### DNRX interacts with DPix via Scribble and modulates the activity of Rac1 to sustain the aggregation and release of SVs

**Figure 6.**
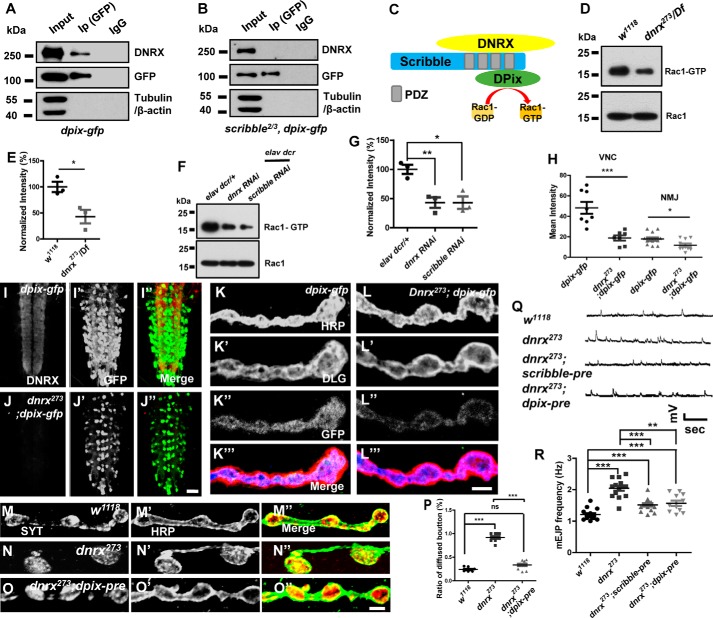
**DNRX interacts with DPix through Scribble and regulates the activity of Rac1, and maintains the terminal SV aggregation and release.** *A,* Western blots of anti-GFP immunoprecipitates using protein lysates of adult *Drosophila* heads showing immunoprecipitated proteins for DNRX and DPix *in vivo*. Input is 1%. *B,* Western blots of anti-GFP immunoprecipitates using protein lysate of adult *Drosophila* heads showing loss of Scribble, DNRX could not immunoprecipitate with DPix *in vivo*. Input is 1%. *C,* schematic representation of DNRX interacts with DPix through Scribble and activates Rac1. *D,* Western blots showing the level of Rac-GTP and total Rac1 in adult heads of *wild-type*, *dnrx* mutant. *E,* summary graph showing that the activated Rac1 was decreased after knocking out DNRX. *F,* Western blots showing the level of Rac-GTP and total Rac1 in adult heads of *elav dcr*/+, *dnrx*, and *scribble* knocking down flies. *G,* summary graph showing that the activated Rac1 was decreased after knocking down the expression of DNRX and Scribble using the RNAi method. *H,* summary graph showing that the mean fluorescence intensity of DPix-GFP was decreased both in NMJ and VNC in *dnrx* mutant when compared with *wild-type* controls. *I* and *J*″, representative images of larvae VNC of the indicated genotypes were stained with DNRX (*red*) and GFP (*green*), showing DNRX regulates the amount of DPix. *K* and *L*‴, representative images of larvae NMJ type Ib boutons of the indicated genotypes were stained with HRP (*blue*), DLG (*red*), and GFP (*green*), showing DNRX regulates the amount of DPix. *M–O*″, confocal images of third instar larvae NMJ type Ib boutons of the indicated genotypes double labeled with anti-SYT (*red*) and anti-HRP (*green*), which labels the pre-synaptic SVs and neuronal membrane, respectively. *P,* quantification of the ratio of the SYT-diffused boutons in muscle 4 shows that in *dnrx* mutants the distribution of SVs has been disturbed and this phenotype can be rescued by pre-synaptic DPix. *Q,* representative traces of spontaneous responses of the indicated genotypes. *R,* quantification of mEJP frequency of indicated genotypes, showing that mEJP frequency was increased in the *dnrx* mutant and the Scribble and DPix could rescue the defects at pre-synapse. Data are mean ± S.E., ***, *p* < 0.001; **, *p* < 0.01; and *, *p* < 0.05. *ns*, not significant. Two-tailed Student's *t* tests were used to compare genotypes. *Scale bars*, 50 (*I–J*″), 5 (*K-L*‴), and 5 μm (*M–O*″).

Mammalian Scribble forms a tight complex with βPix (40), which is a GEF that is involved in the pathways that regulate actin polymerization (42). Based on this, we hypothesized that DNRX regulates presynaptic F-actin through DPix. There is only one DPix in *Drosophila,* whereas two isoforms, αPix and βPix, are found in mammals (43). To reveal the biological role of DPix in DNRX-dependent SV distribution and release, we prepared GFP-fused DPix transgenic flies and overexpressed GFP-DPix in pan-neurons driven by Elav-Gal4. DNRX co-immunoprecipitated with DPix in the *Drosophila* central nervous system (Fig. 6*A*), and this suggested that Scribble might act as a bridge between DNRX and DPix. This hypothesis was supported by another co-immunoprecipitation experiment in which DNRX was not observed in the *scribble* mutants using a GFP antibody (Fig. 6*B*). Therefore, DNRX interacts with DPix through Scribble *in vivo* (Fig. 6*C*), and this indicates that DNRX might regulate presynaptic F-actin via the GEF (DPix).

To further confirm the role of DNRX in presynaptic actin arrangement, we measured the level of activated Rac1, which has been shown to positively promote the polymerization of actin (44, 45). A previous study has shown that Rac1 is locally activated in dendritic spines and that this local activation of Rac1 is regulated by βPix, a Rac GEF (46). Our results showed that DPix associates with DNRX and Scribble. Therefore, we hypothesized that DNRX regulates the activity of Rac1 through its interactions with DPix and thus that the DNRX–Scribble–DPix complex promotes presynaptic actin polymerization. To directly test this, we used GST-fused Pak-PBD, a domain of Pak that can specifically bind to Rac1 and efficiently pulldown activated Rac1 (Rac1-GTP) after treating with GTPrs. Compared with *wild-type*, less Rac1-GTP was pulled down in the *dnrx* mutant (Fig. 6, *D* and *E*). In addition, reducing the expression of DNRX and Scribble in the central nervous system using a RNAi method inhibited the activity of Rac1 (Fig. 6, *F* and *G*). To further confirm whether DNRX regulates actin polymerization and SV release via activating Rac1, we got the dominant-negative form of Rac1 (Rac1^DN^) and integrated it into the *dnrx* mutant background. The immunostaining and electrophysiology results showed that Rac1^DN^ could not rescue the defects of F-actin assembly and SV release in *dnrx* mutant (supplemental Fig. S5). Similarly, we measured another GTPase signaling protein, Cdc42, which can also regulate actin cytoskeleton like Rac1 (47, 48), however, the results showed that the level of activated Cdc42 has no significant difference between *wild-type* and *dnrx* mutant although it has a decreased tendency in *dnrx* mutant (supplemental Fig. S6).

To further determine whether DNRX can affect the distribution and level of pre-synaptic DPix, as mentioned above, the lack of DPix antibody and the fact that DPix is localized at both the pre- and postsynapse (49) forced us to use motor neuron-specific Gal4 (OK6-Gal4) to drive DPix in the *dnrx* mutant and *wild-type* background separately. Surprisingly, we found that the level of DPix was significantly decreased in the *dnrx* mutant compared with *wild-type* background both in the NMJ and VNC (Fig. 6, *I–L*‴), and the fluorescence intensity measurements validated this phenotype (Fig. 6*H*). To determine whether the level of DPix mRNA was also changed in the *dnrx* mutant, we performed qRT-PCR and found that the mRNA level of DPix had a downward tendency, but the differences compared with controls were not statistically significant (supplemental Fig. S3*A*). Collectively, these results highlight the essential role of DNRX in affecting GEF through Scribble and support the hypothesis that DNRX regulates presynaptic F-actin by activating Rac1.

To determine whether SV distribution is altered in *dpix* knockdown flies, we examined the terminal SVs in *dpix RNAi* driven by elav-gal4 and we found that SVs are diffused in bouton after knocking down the expression of DPix (supplemental Fig. S3, *B–D*″). To further confirm the functional significance of DPix in DNRX-dependent aggregation of SVs, we next independently overexpressed DPix in the *dnrx* mutant background and the immunostaining result showed that the ratio of SYT-diffused boutons could be restored to the *wild-type* level (Fig. 6, *M–O*″ and *P*). However, the defect in which SVs are retained in the axon in the *dnrx* mutant could not be rescued by Scribble–DPix (supplemental Fig. S4, *A–D*″). To better understand the effect of Scribble–DPix on DNRX, we assessed the spontaneous release of SVs and found that Scribble–DPix can rescue the abnormality in the *dnrx* mutant (Fig. 6, *Q–R*).

### The PDZ-binding motif is essential for the effect of DNRX on F-actin assembly and SV release

**Figure 7.**
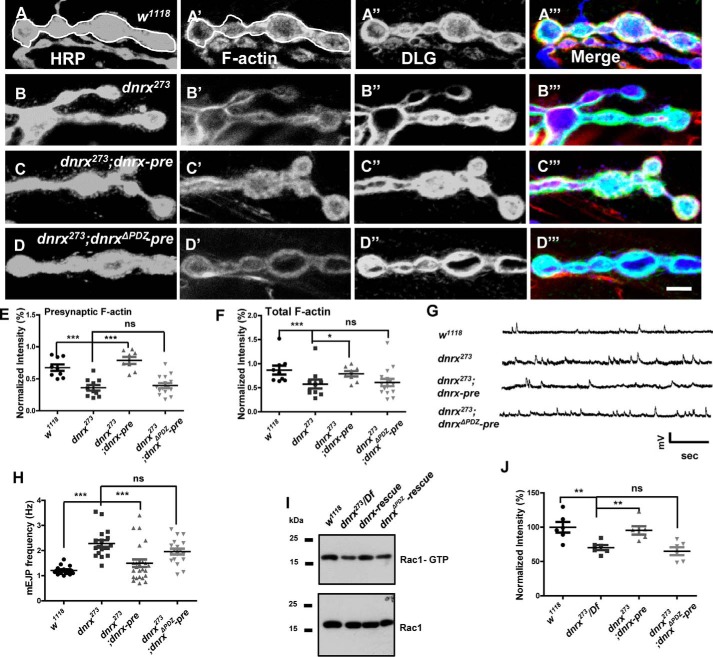
**The PDZ-binding motif is essential for the effect of DNRX on F-actin assembly and SV release.** *A–D*‴, representative images of third instar larvae NMJ type Ib boutons at muscles 12/13 triple labeled with Texas Red phalloidin (*red*), anti-DLG (*green*), and anti-HRP (*blue*) in *wild-type* (*A*), *dnrx* mutants (*B*), *dnrx* rescue (*C*), and *dnrx*^Δ*PDZ*^ rescue (*D*). The phalloidin signal contained in *white circles* corresponds to HRP, and largely reflects the presynaptic F-actin. *E* and *F,* summary graph of the relative fluorescence intensity of F-actin corresponding to HRP (*E*) and DLG (*F*) showing that F-actin fluorescence intensities were significantly reduced in *dnrx* mutant and could not be rescued by inducing DNRX^ΔPDZ^ at presynapse. *G,* representative traces of spontaneous responses of the indicated genotypes. *H,* quantification of mEJP frequency of the indicated genotypes, showing that mEJP frequency was increased in the *dnrx* mutant and that DNRX^ΔPDZ^ could not rescue the defects. *I,* Western blots showing the level of Rac-GTP and total Rac1 in adult heads of *wild-type*, *dnrx* mutant, *dnrx* rescue, and *dnrx*^Δ*PDZ*^ rescue. *J,* summary graph showing that the activated Rac1 was decreased after knocking out DNRX and this defect can be rescued by inducing the full-length of DNRX at presynapse, however, driving DNRX^ΔPDZ^ at presynapse in the *dnrx* mutant had no rescue effect. Data are mean ± S.E. ***, *p* < 0.001; **, *p* < 0.01; and *, *p* < 0.05. *ns*, not significant. Two-tailed Student's *t* tests were used to compare genotypes. *Scale bar*, 5 μm (*A–D*‴).

**Figure 8.**
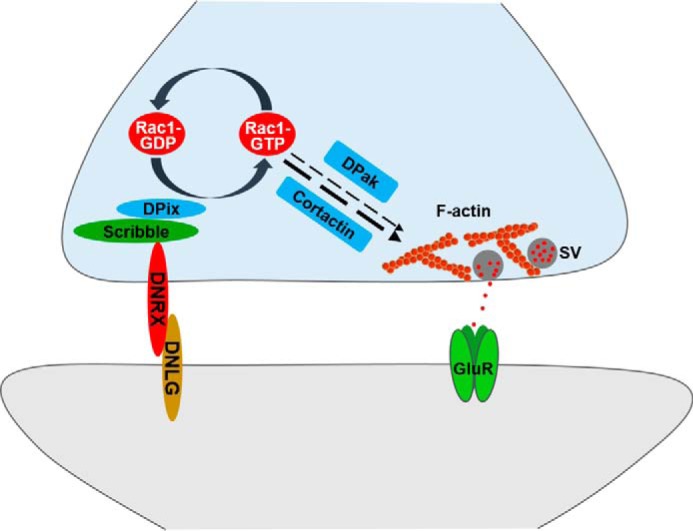
**The model shows that DNRX regulates SV aggregation and release via presynaptic F-actin by modulating Scribble–DPix.** Presynaptic DNRX interacts with Scribble–DPix to regulate the activity of Rac1, the activated Rac1 (*Rac1-GTP*) then affects actin polymerization to finally modulate the SVs localization and release.

To determine the specific motif of DNRX that might be involved in the regulation of F-actin and SV, we subsequently constructed the *dnrx*^Δ*PDZ*^ transgenic fly, which lacks the C-terminal 7 amino acid residues according to the above result. To determine whether the PDZ-binding motif of DNRX is required for F-actin assembly, we first examined the F-actin intensity in *wild-type*, *dnrx* mutant, *dnrx* rescue, and *dnrx*^Δ*PDZ*^ rescue lines. The immunostaining results showed that the fluorescence intensity of F-actin was significantly reduced in *dnrx* mutants and this defect could not be restored to normal levels after introducing DNRX^ΔPDZ^ at presynapse (Fig. 7, *A–D*‴). Furthermore, we measured the fluorescence intensity of F-actin in relationship to neuronal plasma membrane (HRP) and Discs large (DLG). The data showed that DNRX^ΔPDZ^ had no effect on the regulation of F-actin (Fig. 7, *E* and *F*). Next, to examine the association of the DNRX PDZ-binding motif with SV release, we performed electrophysiology experiments and the result also showed that DNRX^ΔPDZ^ could not rescue the increased frequency of SV release observed in the *dnrx* mutant (Fig. 7, *G* and *H*). Finally, to better understand the effect of DNRX on the activity of Rac1, we assessed the activated Rac1 level after inducing DNRX^ΔPDZ^ in the *dnrx* mutant background and we found that DNRX^ΔPDZ^ still could not rescue the defect of Rac1 activity (Fig. 7, *I* and *J*). Altogether, our data consistently indicate that the PDZ-binding motif of DNRX is essential for regulating F-actin assembly and SV release. Therefore, our results support a model that DNRX binds to DPix via Scribble and regulates F-actin polymerization by activating Rac1, and subsequently control SV clustering and release at synaptic terminals (Fig. 8).

## Discussion

The brain is one of the most complicated organs and synapse is the functional unit of the brain. A large number of genes and proteins are involved in regulating the function of the brain. Mutations in synapse-associated genes cause neurologic disease. It would thus be desirable to explore the fundamental function of these genes *in vivo* and it may help attenuate the suffering of the patient. Considering the genetic techniques are available to manipulate, rapid life cycle, low cost, and a large number of genes are conserved between flies and vertebrates, it makes *Drosophila* emerging as an outstanding model to identify the biological basis for disease. Here, we use *drosophila* NMJ as a model to study and reveal the vital function of the cell adhesion molecule DNRX at synapse. Our study provides new mechanistic insights into the SV cluster and release regulation, which conceptually advances current knowledge of DNRX function at synapse.

### DNRX and Scribble are essential for nervous system

Neurexin is a highly conserved cell adhesion molecule that is predominantly localized at the presynaptic terminal. Previous studies have shown that Neurexin plays a significant role in synaptic architecture and function. In addition, accumulating evidence has implicated Neurexin in ASDs. ASDs are neurodevelopmental disorders characterized by deficits in communication and social interaction as well as restricted interests and repetitive and stereotypic patterns of behavior (10, 20, 50, 51). However, the precise function and underlying molecular mechanisms of Neurexin in both normal physiology and ASDs remain unclear. *Drosophila* Scribble is a cytoplasmic scaffolding protein that was first recognized as a tumor suppressor that regulates epithelial cell adhesion and migration in mammals (31, 32). Recently, it has been shown that Scribble is also localized in the nervous system both in invertebrate and vertebrate animals (33, 41), and plays a role in synaptic plasticity and animal behavior, including learning, memory, social behavior, and olfactory behavior (52). However, how Scribble functions at the synapse remains unknown. In this study, we have revealed that DNRX interacts directly with the Scribble PDZ domains through the very C-terminal PDZ-binding motif to regulate presynaptic F-actin and SVs.

### F-actin is highly enriched at synaptic terminals and is vital for SV traffic, localization, and release

For decades, F-actin emerged as the major cytoskeleton identified in presynaptic nerve terminals. Considering the especial location of F-actin at the internal space of presynaptic nerve terminals, it raised a hypothesis that F-actin regulates synaptic vesicle localization and release. Previous studies from several groups provided consistent evidence that after disrupting the polymerization of actin, pre-synapse affected SV traffic, localization, and neurotransmitter release (4, 5, 28). Moreover, cortical actin has been identified as a barrier for vesicles during the process of moving to the active zone in the presynaptic terminal (27). However, whether and how F-actin participates in the regulation of SV at synapse is still controversial. Therefore, further investigation will be necessary to determine the function of F-actin at synapse and especially for advances in our understanding of the relationship with SV. By now the function of F-actin at synapse is still poorly understood. To better understand the effect of F-actin on SV we assessed the cluster and release of SV after ablating the actin-associated genes and found the obvious defects of SV cluster and release. To further test this possibility, we need to perform additional experiments to elaborate the vital role of F-actin in SV regulation in future.

### The function of DNRX in regulating SVs via presynaptic F-actin

SV distribution and dynamics are essential for normal neural signal transmission and for synaptic plasticity both at peripheral and central synapses (53). Disruptions in SV function will lead to various forms of neurological disorders. The process of synaptic vesicle priming, docking, and fusion with the presynaptic membrane has been investigated extensively, and numerous molecules involved in this process have been identified. These include synaptotagmins, synapsins, synaptobrevins, and Munc18. However, how SV cluster in particular compartments and their role in regulating neurotransmitter release at the presynaptic terminal are unclear. The results from this study provide compelling evidences that DNRX plays an essential role in the distribution and release of SVs.

In this study, our data suggest the amount of F-actin is significantly reduced. Moreover, presynaptic Cortactin or the active form of DPak, key regulators of the actin cytoskeleton, are able to rescue the defects in SV distribution and spontaneous release frequency in the *dnrx* mutant. These results illustrate the essential role of presynaptic actin in regulating SVs localization and release. Our results are consistent with the recent findings showing that Arp2/3 complex-mediated actin regulation is important for presynaptic neurotransmitter release (54).

### Scribble is required for DNRX associates with DPix

Previous work has shown that Scribble is a scaffolding, tumor suppressor protein that through its PDZ domains interacts with a number of proteins, including β-catenin, βPix, and NOS1AP. Although NOS1AP can bind directly to the fourth Scribble PDZ domain (41), βPix has binding affinity to all four PDZ domains of Scribble (40). In the present study, we use co-immunoprecipitation to show that DNRX can form a complex with Scribble in the *Drosophila* nervous system *in vivo*. We also show that DNRX and Scribble are co-localized in both central and peripheral nervous system during embryonic, larval, and adult stages. Importantly, these two proteins are highly expressed in the mushroom body of *Drosophila*, suggesting a key role in learning and memory. We also show that DNRX can directly bind to all four Scribble PDZ domains through its C-terminal PDZ-binding motif, similar to what is observed for βPix. These interaction results suggest that there may be competitions and cross-effect between DNRX and βPix. These interactions may also relate to the mutual effect on the protein level of Scribble and DNRX. Because the mRNA level of Scribble is not altered in the *dnrx* mutant, it is possible that when the DNRX or Scribble was absent, the complex becomes destabilized and subsequently degraded. Further experiments are needed to address this possibility.

### DNRX–Scribble–DPix regulates presynaptic F-actin by activating Rac1

What is the functional consequence of the Scribble and DNRX interaction? The present study provides evidence that Scribble may act as a bridge between DNRX and DPix to regulate the actin cytoskeleton. βPix is a GEF specific to Rac1/Cdc2, a key mediator of actin reorganization in response to various stimuli. In the mammalian system, Rac1 is locally activated in dendritic spines, and this spatial restricted activation is regulated by Pix (46). In the present study, we show that the active form of Rac1 (Rac1-GTP) is reduced in the *dnrx* mutant, *dnrx*, and *scribble* knockdown flies compared with *wild-type* for the protein level of DPix in these lines were decreased, supporting the idea that the DNRX and Scribble interaction activates Rac1. Recent studies show that Rac1 plays a critical roles in animal behavior, particularly in the process of forgetting (36). In addition, Scribble has recently been reported to activate forgetting through Rac1 in *Drosophila* (35). Taken together, we suggest that DNRX may also be involved in forgetting by regulating the Rac1 signaling pathway. Indeed, it has been demonstrated that Rac1 activation is defective in multiple autism-related gene mutations, including the *dnrx* gene, and that Rac1 has been proposed to be a converging node linked to ASD (36). Thus, the present study demonstrating that DNRX interacts with Scribble and DPix to regulate Rac1 provides direct mechanistic insight into not only the fundamental mechanisms underlying the roles of the neuroligin–neurexin complex in actin-mediated presynaptic regulation, but also the pathological mechanism of ASD.

## Experimental procedures

### cDNA clones

The Scribble, DPix, Cortactin, and DNRX full-length cDNA were obtained from DGRC.

### Fly stocks

The following fly strains were used in this study. 1) The *dnrx* mutant allele *dnrx*273 was generated as a gift from Dr. Manzoor A. Bhat, and Df(3R)5C1 (a deficiency that removes *dnrx*) were introduced into the TM6B fly line that was obtained from the Bloomington Stock Center. The *dnrx* mutant allele *dnrx*83 was generated by our lab (9). UAS-*dnrx* and *dnrx*^Δ*PDZ*^ transgenic flies were generated by cloning the entire *dnrx* full-length cDNA and *dnrx* full-length cDNA without the terminal region of the PDZ-binding motif into the pUAST-attB vector for germ line transformation. 2) Mutations in *scribble, scribble2*, and *scribble3* were generated as a gift from Norbert Perrimon, and Df(3R)Tl-x flies, which are missing the *scribble* region, were obtained from the Bloomington Stock Center. UAS-scribble-gfp and UAS-dpix-gfp transgenic flies were generated by cloning the entire scribble, dpix full-length cDNA with GFP cDNA C-terminal into the pUAST-attB vector for germ line transformation. 3) The Gal4 lines, Elav-Gal4, Elav dcr-Gal4, and OK6-Gal4 were obtained from the Bloomington Stock Center. 4) The UAS-dnrx RNAi, UAS-scribble RNAi, UAS-dpix RNAi, UAS-actin-GFP transgenic fly, and scribble::gfp (07683) were purchased from the model animal center of *D. melanogaster* Tsinghua University. The uas-pak, usa-rac^L89^ (dominant-nagtive rac), and *tsr* mutant flies were obtained from the Bloomington Stock Center. 5) The *wild-type* (WT) *D. melanogaster* strain used in this study was *w^1118^*. 6) All transgenic flies and gene Integrated flies were identified (supplemental Fig. S7). All the flies were raised at 25 °C on standard medium.

### Western blotting analysis

Western blotting analysis was performed as described previously (9). In brief, adult heads and larvae muscles were homogenized with RAPI buffer and centrifuged at 12,000 rpm at 4 °C for 10 min, and the supernatant was collected. The protein lysates were electrophoresed on an SDS-PAGE gel and electrotransferred onto polyvinylidene difluoride membranes. Immobilized proteins on the membrane were probed with the primary antibody and then incubated with HRP-conjugated secondary antibodies. The primary antibodies used in this study were rabbit polyclonal anti-DNRX (55); mouse monoclonal anti-GFP, mouse anti-Arp2, and mouse anti-Cortactin from Santa Cruz Biotechnology; mouse monoclonal anti-α-tubulin and anti-β-actin were from Sigma; mouse anti-DLG, mouse anti-Brp, and mouse anti-Synapsin antibodies from the Developmental Studies Hybridoma Bank; rabbit polyclonal anti-Cdc42 from Thermo Scientific.

### Immunostaining

Preparation and antibody staining for whole mount embryos, wandering third-instar larvae, and adult. Briefly, embryonic samples were washed three times in 0.3% PBST (PBS + 0.3% Triton X-100) rapidly, adding 50% commercial bleach diluted in water for decorticating, blocked for 1 h in the blocking solution, and incubated with primary antibodies at 4 °C overnight. For larvae and adult NMJ and head staining, larvae and adult were dissected in Ca^2+^-free HL3 saline (70 mm NaCl, 5 mm KCl, 20 mm MgCl_2_, 10 mm NaHCO_3_, 5 mm trehalose, 115 mm sucrose, and 5 mm Hepes, pH 7.2) and fixed in 4% paraformaldehyde/PBS for 30 min. After dissection and fixation, samples were stained with antibodies. The primary antibodies used in this study were as follows: purified mouse antiserum against DNRX (A4, 1:100) (14), mouse anti-SYT (DSHB, 1:50), mouse anti-SYN (DSHB, 1:50), mouse anti-DLG (DSHB, 1:50), mouse anti-GFP (DSHB, 1:50), and rabbit anti-HRP (DSHB, 1:500), Texas Red®-X Phalloidin (Molecular Probes, 1:10) and Alexa Fluor 488-, Alexa Fluor 555-, or Alexa Fluor 647-conjugated secondary antibodies (Invitrogen, 1:500). Samples were imaged at room temperature using a LSM 700 (Carl Zeiss) confocal microscope at 1,024 × 1,024 pixels using a ×63 (NA 1.4, oil immersion; Carl Zeiss) objective for boutons staining, ×40 (NA 0.95, dry; Carl Zeiss) objective for whole NMJ staining, ×20 (NA 0.8, dry; Carl Zeiss) for epithelial cells staining and ×10 (NA 0.45, dry; Carl Zeiss) for brain, VNC, and embryo staining. All images were initially acquired through Zen 2010 software (Carl Zeiss). Fluorescent intensities for F-actin, DNRX, Scribble::GFP, and DPix-GFP were measured using Image J software (National Institutes of Health). Brightness and contrast were adjusted using AimImageBrowser software (Carl Zeiss).

### STED imaging

For STED imaging the Alexa Fluor 555-conjugated secondary antibodies were used (Invitrogen, 1:500). Samples were imaged at room temperature using a STED microscope of TCS SP8 (Leica, Germany) at 1,024 × 1,024 pixels using a ×100 (NA 1.4, oil immersion; Leica) objective for boutons staining. All images were initially acquired through LAS X software (Leica). All images were treated with Huygens Professional software.

### Immunoprecipitation

The immunoprecipitation experiments were performed as previously described (56). Briefly, fly heads of the desired genotypes were homogenized using a glass homogenizer in ice-cold lysis buffer containing 20 mm Tris (pH 7.5), 150 mm NaCl, 1% Triton X-100, and sodium pyrophosphate, β-glycerophosphate, EDTA, Na_3_VO_4_, and leupeptin (Beyotime Biotechnology) with protease inhibitors. The lysates were kept on a shaker at 4 °C for 30 min and centrifuged twice at 12,000 rpm at 4 °C for 10 min. The supernatant was incubated with beads and antibody on a shaker at 4 °C overnight. Then centrifuged at 2,500 rpm at 4 °C for 2 min and the supernatant was discarded. The beads were subsequently washed three times with lysis buffer, and proteins were eluted by boiling the beads in 2× SDS loading buffer. Then centrifuged at 12,000 rpm at room temperature for 2–3 min and the beads were discarded, the remnant buffer was used for the immunoblot analysis.

### GST pulldown assay

The methods for the GST pulldown assay has been described previously (57). Briefly, GST fusion proteins were expressed in BL21 *Escherichia coli* cells with pGEX-5X-1 vector, and then purified and immobilized with glutathione-Sepharose 4B beads (GE Healthcare). Plasmids coding the four Scribble PDZ domains of pCDNA3.1 were transfected into HEK293T cells. After inducing and expressing, transfected cells were lysed in the lysis buffer (Roche Applied Science) and incubated with beads that contained GST-fused protein. The beads were subsequently washed three times with lysis buffer, and proteins were eluted by boiling the beads in 2× SDS loading buffer. The beads were then centrifuged at 12,000 rpm at room temperature for 2–3 min and the beads were discarded, the remnant buffer was used for immunoblot analysis.

### Cell culture, transfection, and treatments

DMEM (Sigma) was used to culture HEK293 and supplemented with 10% FBS (Invitrogen). Cells were transfected with X-tremeGENE 9 DNA Transfection Reagent (Roche Applied Science) according to the manufacturer's protocols and grown in a single Petri dish (90 × 90 mm). After incubating and maintaining the cells for 48 h at 37 °C, they were harvested for biochemical analyses.

### Electrophysiology

Third instar larvae were dissected as described previously (58). Briefly, third instar larvae were rapidly dissected in Ca^2+^-free HL3.1 saline, fat and gut in the body were removed, brain and VNC were cut, and the body wall was carefully spread out to expose muscle to avoid damage to the muscle architecture. The free segmental nerve end was drawn into a microelectrode using a injector and stimulated with a Grass S48 stimulator (Astro-Grass) at 0.3 Hz with a suprathreshold stimulating pulse, and we selected NMJ 6 in the A3 segment for recording using recording electrodes (20–50 MΩ) filled with 3 m KCl. mEJPs were recorded, the start was from 8 s after EJP recordings for 20 s. All recordings were conducted in a modified HL3.1 solution containing 70 mm NaCl, 5 mm KCl, 4 mm MgCl_2_, 10 mm NaHCO_3_, 0.6 mm CaCl_2_, 115 mm sucrose, 5 mm trehalose, and 5 mm Hepes (pH 7.2). Recordings were performed at room temperature with an Axoclamp 900A amplifier (Molecular Devices, Sunnyvale, CA) in bridge mode. Recording data were digitized with a Digitizer 1322A (Molecular Devices). Data were analyzed in the Clampfit 10.2 software.

### Rac1 activity assay

The heads of ∼500 adult flies were isolated and ground to gain sample. For detecting the relative levels of Rac1-GTP, a Pak-PBD pulldown assay (no. 16118; Thermo Scientific) was used according to the manufacturer's procedures as described previously (36). The anti-human Rac1 monoclonal antibody (1:1,000 dilution) was used to detect total and activated Rac1 levels.

### Statistical analysis

Statistical significance was determined using Student's *t* tests for comparisons of two groups and one-way analysis of variance followed by the appropriate post hoc test for comparisons of multiple group means (GraphPad Prism 5). All *asterisks above a line* indicate comparisons between the two groups indicated. Data are expressed as the mean ± S.E. *p* values <0.05 were considered to be statistically significant.

## Author contributions

W. X. conceived the project, W. X. and M. L. R. designed the experiments and wrote the manuscript, W. X., M. L. R., J. H. H., and Z. P. J. analyzed data. M. L. R. conducted the experiments. J. J. Q. performed the qRT-PCR experiment. L. J. L., Y. H. C., and H. H. L. helped in recoding and animal analysis.

## Supplementary Material

Supplemental Data

## Footnotes

2 The abbreviations used are:

SV: synaptic vesicle
ASDs: autism spectrum disorders
AZ: active zone
BRP: bruchpilot
DLG: Discs large
DNRX: *Drosophila* Neurexin
DPix: *Drosophila* Pix
EJP: excitatory junction potential
GEF: guanine nucleotide exchange factor
HRP: neuronal plasma membrane
mEJP: miniature excitatory junction potentials
NMJ: neuromuscular junction
SYN: synapsin
SYT: synaptotagmin
VNC: ventral nerve cord
STED: stimulated emission depletion
qRT: quantitative RT.
