# Supplementary material for: The neuronal protein Neurexin directly interacts with the Scribble–Pix complex to stimulate F-actin assembly for synaptic vesicle clustering
Source: J Biol Chem. 2017 Jul 14;292(35):14334–48. doi: 10.1074/jbc.M117.794040 (PMC5582829; doi:10.1074/jbc.M117.794040)
Supplement: Supplemental Data [file supp_292_35_14334__index.html]

The Neuronal Protein Neurexin Directly Interacts with the Scribble-Pix Complex to Stimulate F-actin Assembly for Synaptic Vesicle Clustering — The neuronal protein Neurexin directly interacts with the Scribble–Pix complex to stimulate F-actin assembly for synaptic vesicle clustering — Neurexin clusters synaptic vesicles — Supplemental Data 

# The neuronal protein Neurexin directly interacts with the Scribble–Pix complex to stimulate F-actin assembly for synaptic vesicle clustering

## Supplemental Data

- allowable supplemental data (.pdf, 1.4 MB) - This file contains allowable supplemental data
