# Supplementary material for: The neuronal protein Neurexin directly interacts with the Scribble–Pix complex to stimulate F-actin assembly for synaptic vesicle clustering
Source: J Biol Chem. 2017 Jul 14;292(35):14334–48. doi: 10.1074/jbc.M117.794040 (PMC5582829; doi:10.1074/jbc.M117.794040)
Supplement: Supplemental Data [file 10.1074_M117.794040_jbc.M117.794040-1.pdf]

The Neuronal Protein Neurexin Directly Interacts with the Scribble-Pix Complex to Stimulate F-actin Assembly for Synaptic Vesicle Clustering

Menglong Rui[1], Jinjun Qian[1], Lijuan Liu[1], Huihui Lv[1], Junhai Han[1,2], Zhengping Jia[3,4] and Wei Xie[1,2]*

SUPPLEMENTAL DATA

Figure S1

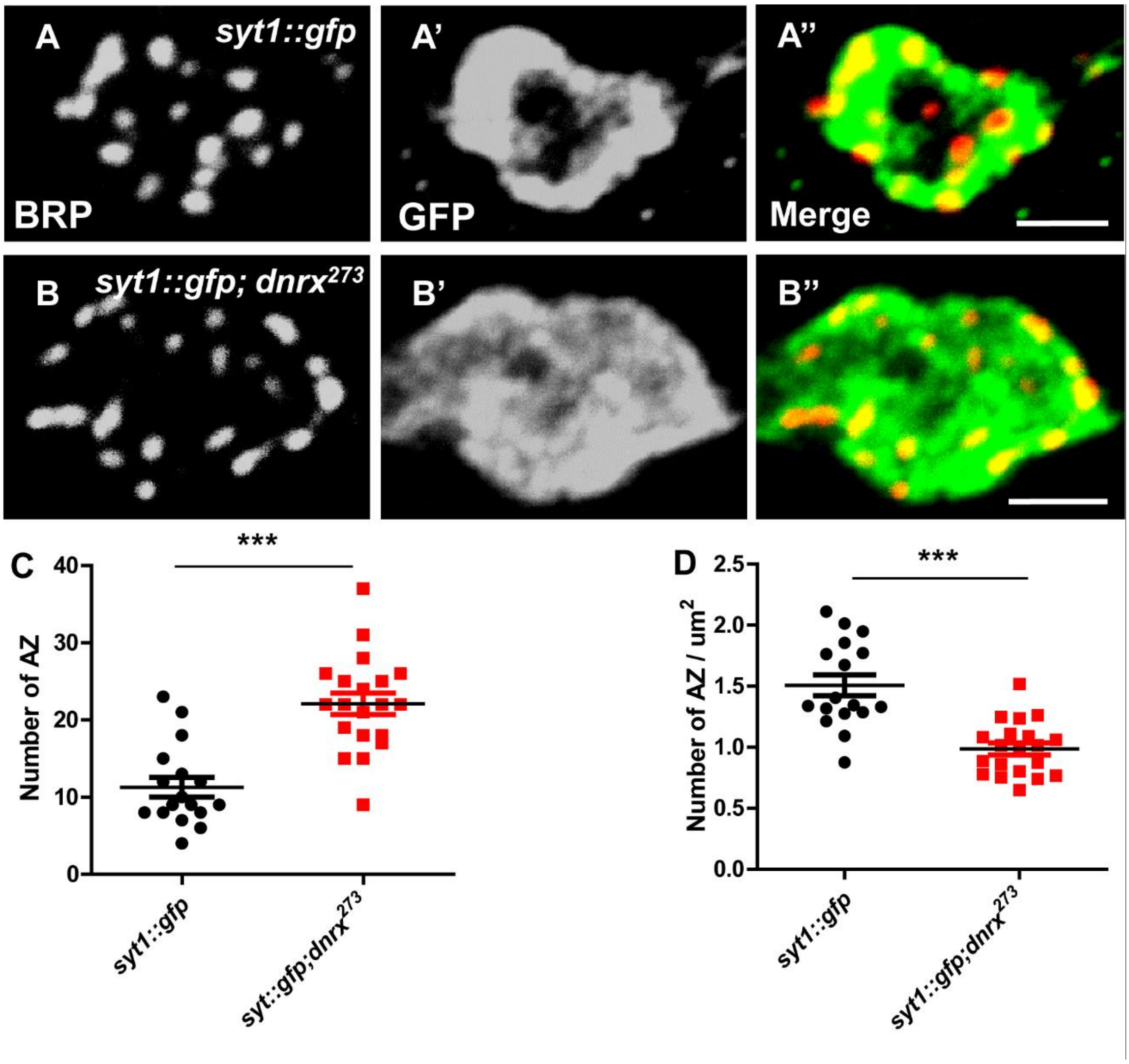

Figure S1. The Number and Density of AZs Are Altered in *dnrx* Mutant. (A-B'') Confocal images of third instar larvae single bouton double labeled with anti-BRP (red), anti-GFP (green) in in *syt::gfp* (A-A''), *syt::gfp;dnrx*^{273} (B-B''), showing that loss of DNRX disrupts the number and density of AZ. (C) Quantification of the number of T-bar in *syt::gfp* and *dnrx*^{273}, *syt::gfp* mutants shows that loss of DNRX disrupts the number of T-bar. (D) Quantification of the density of T-bar in *syt::gfp* and *dnrx*^{273}, *syt::gfp* mutants shows that loss of DNRX disrupts the density of T-bar. Data are mean ± SEM, ***p < 0.001. Two-tailed Student's *t* tests were used to compare genotypes. Scale bar, 2 μm (A-A''), 2 μm (B-

B'').

Figure S2

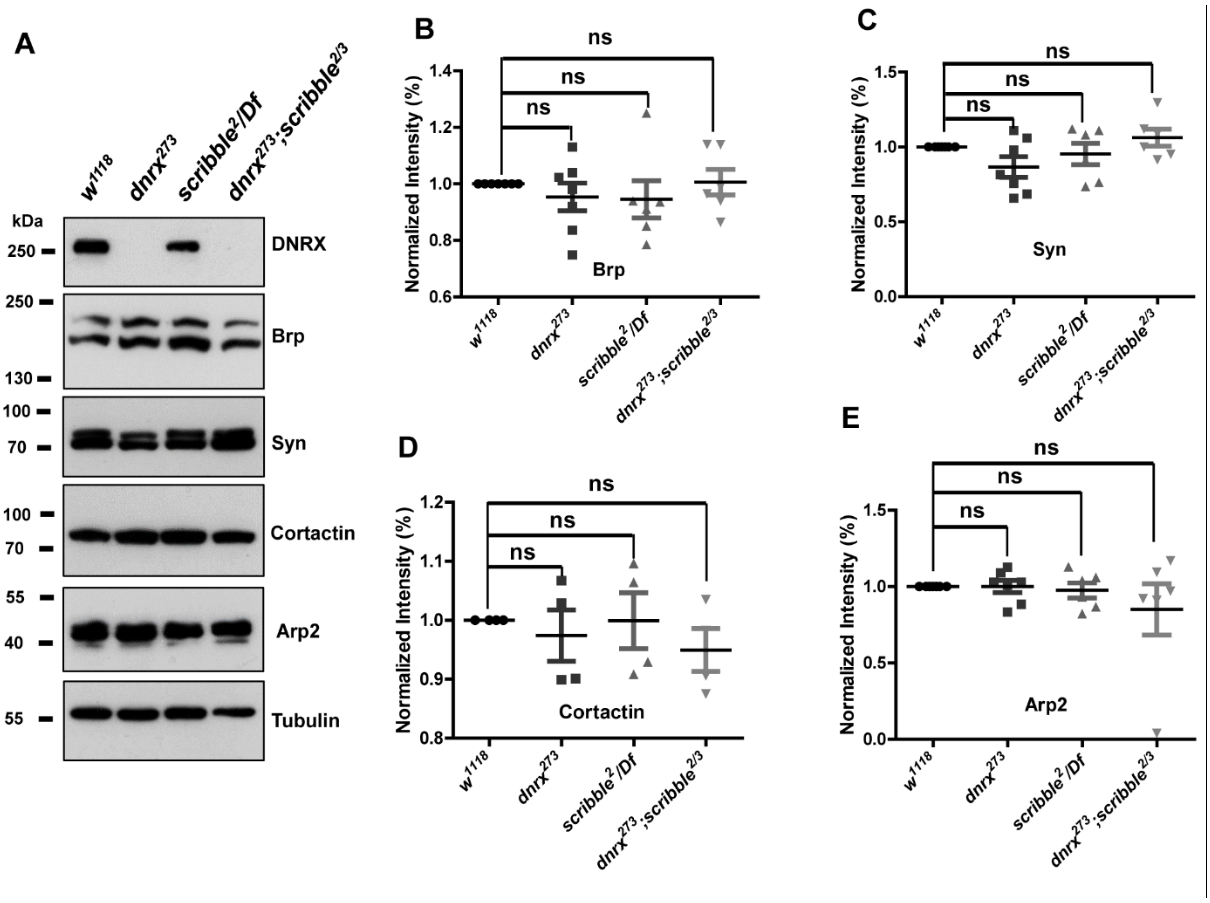

Figure S2. Loss of DNRX and Scribble Do not Affect the Amount of SV, Synapse and F-actin Associated Molecules. (A) Western blot analysis of protein lysates prepared from heads of adult flies using anti-DNRX, Brp, Synapsin, Cortactin, Arp2 and Tubullin antibodies, showing that the expression level of these proteins were similar in *wild-type*, *dnrx*^{273}, *scribble*$^{2/3}$, *dnrx*^{273}*;scribble*$^{2/3}$. (B-E) Summary graphs showing the normalized intensity of proteins for Brp, Synapsin, Cortactin and Arp2 in *wild-type, dnrx*^{273}, *scribble*$^{2/3}$, *dnrx*^{273}*;scribble*$^{2/3}$. ns: not significant. Two-tailed Student's *t* tests were used to compare genotypes.

Figure S3

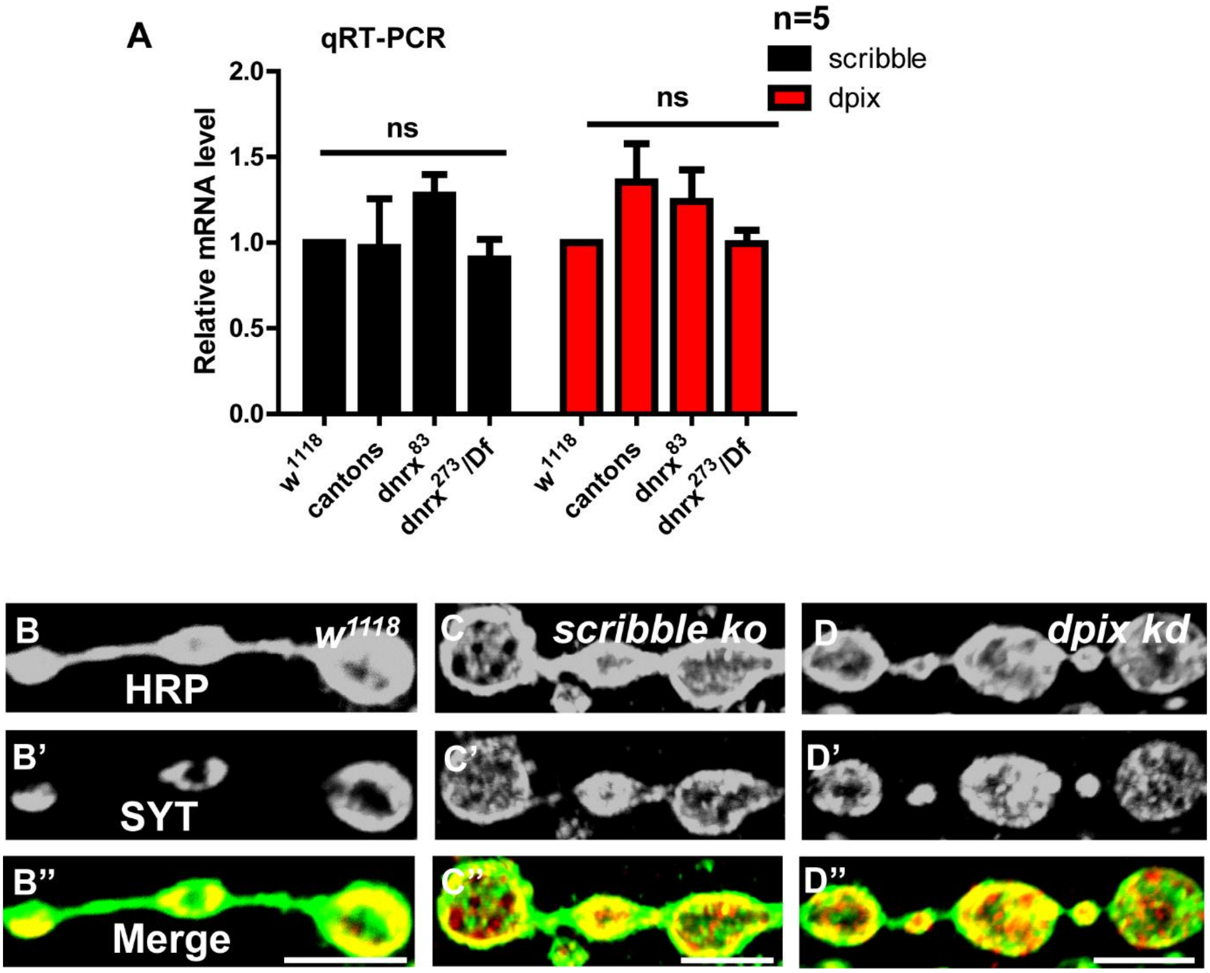

Figure S3. The Scribble/Dpix Complex Is Essential for SV Distribution in Bouton and the RNA Levels of Scribble and DPix in *dnrx* Mutant and *wild-type* Are Similar. (A) qRT-PCR analysis and quantification of the relative mRNA level for Scribble, DPix in *wild-type* and *dnrx* mutant respectively, showing the mRNA level of Scribble and DPix has no change in *dnrx* mutant when compared with control. ns: not significant. One-way ANOVA was used for comparisons of the 4 groups (w^{1118}, *cantons*, $dnrx^{83}$ and $dnrx^{273}/Df$). (B-D'') Three instar larvae NMJ of *wild-typ*e, *scribble ko*, *dpix kd* staining with anti-HRP (green) and SYT(red), showing that the terminal SV distribution is diffused in *scribble ko* and *dpix kd* when compared *with wild-typ*e. Scale bar, 5 µm (B-B''), 5 µm (C-C''), 5 µm (D-D'').

Figure S4

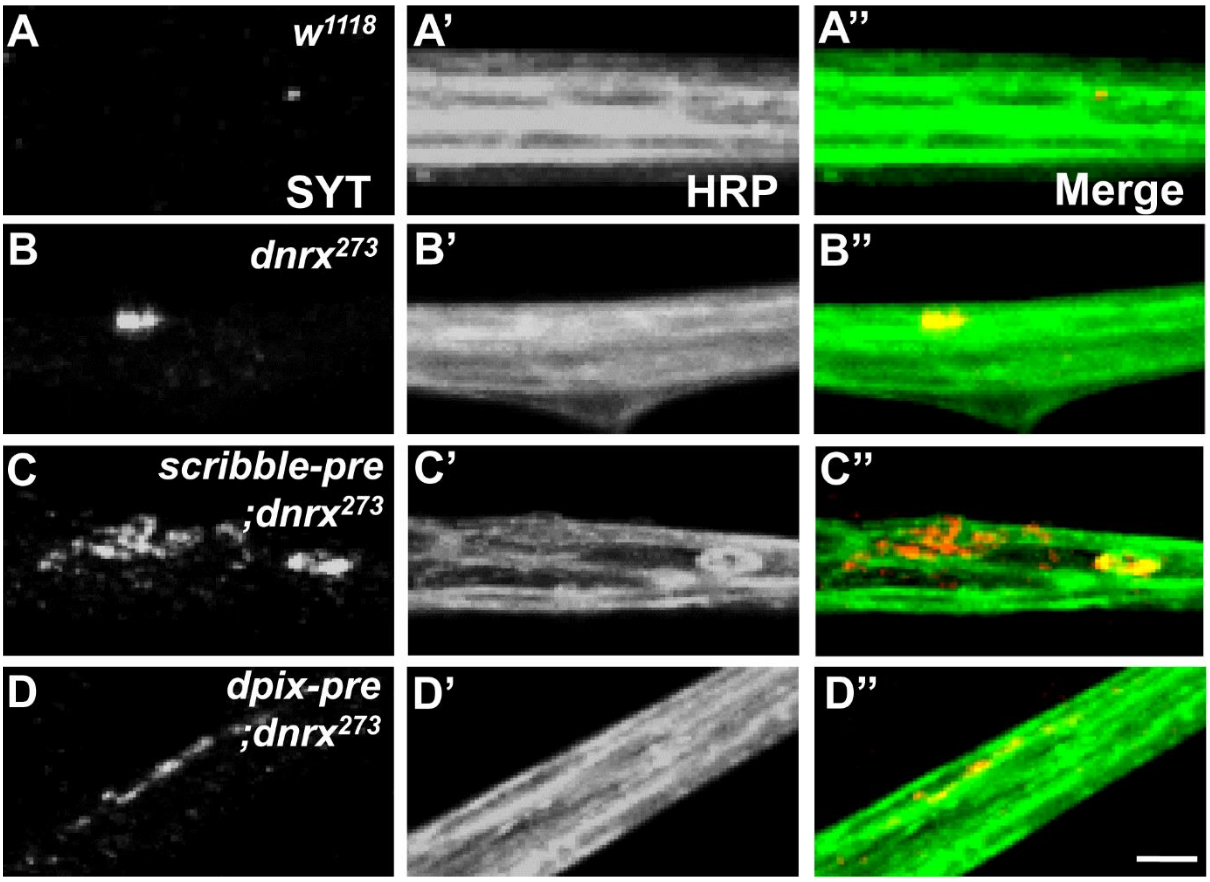

Figure S4. DNRX Regulates SV Transportation in Motor Axon Independent of Scribble/DPix Complex. (A-D'') Confocal images of third instar larvae motor neuron axon labeled with SYT (red), HRP (green) in *wild-type*, *dnrx*^{273}, *ok6>uas-scribble,dnrx*^{273}, *ok6>uas-dpix,dnrx*^{273}, showing the SVs were stranded at axon in *dnrx* mutant and this defect could not be rescued by Scribble and DPix. Scale bar, 10 μm (A-D'').

Figure S5

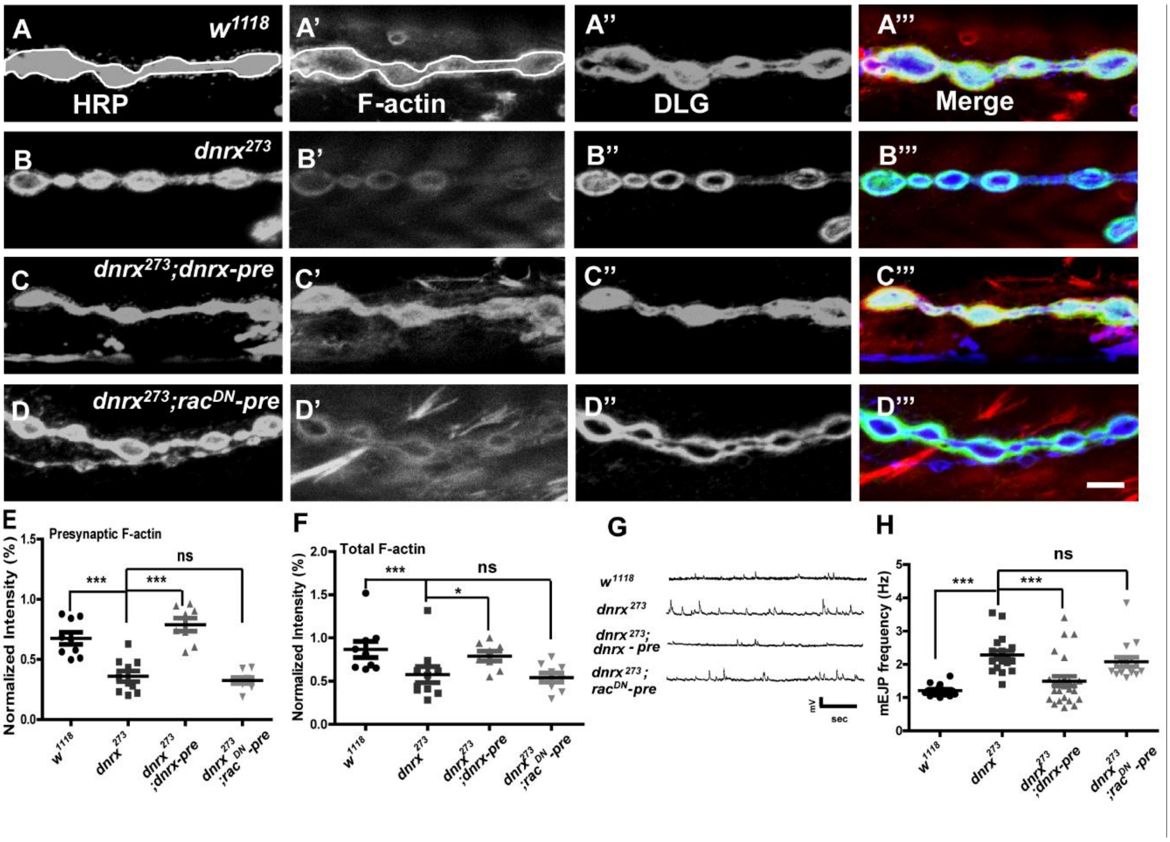

Figure S5. The Dominant Negative Rac1 could not Rescue the Defeat of F-actin Assembly and SV Release in *dnrx* Mutant. (A-D''') Representative confocal images of third instar larvae NMJ type Ib boutons at muscles 12/13 triple labeled with Texas red phalloidin (red), anti-DLG (green) and anti-HRP (blue) in *wild-type* (A), *dnrx* mutants (B), *dnrx* rescue (C), rac^{DN} rescue (D). The phalloidin signal contained in white circles correspond to HRP, largely reflects the presynaptic F-actin. (E-F) Summary graph of relative fluorescence intensity of F-actin correspond to HRP (E) and DLG (F) showing that the F-actin fluorescence intensities were significantly reduced in *dnrx* mutant and could not be restored to normal level after driving Rac^{DN} at presynapse. (G) Representative traces of spontaneous responses of indicated genotypes. (H) Quantification of mEJP frequency of indicated genotypes, showing that mEJP frequency was increased in *dnrx* mutant and the Rac^{DN} could not rescue the defects. Data are mean ± SEM. ***$p < 0.001$ and *$p < 0.05$. ns: not significant. Two-tailed Student's *t* tests were used to compare genotypes. Scale bar, 5 µm (A-D''').

Figure S6

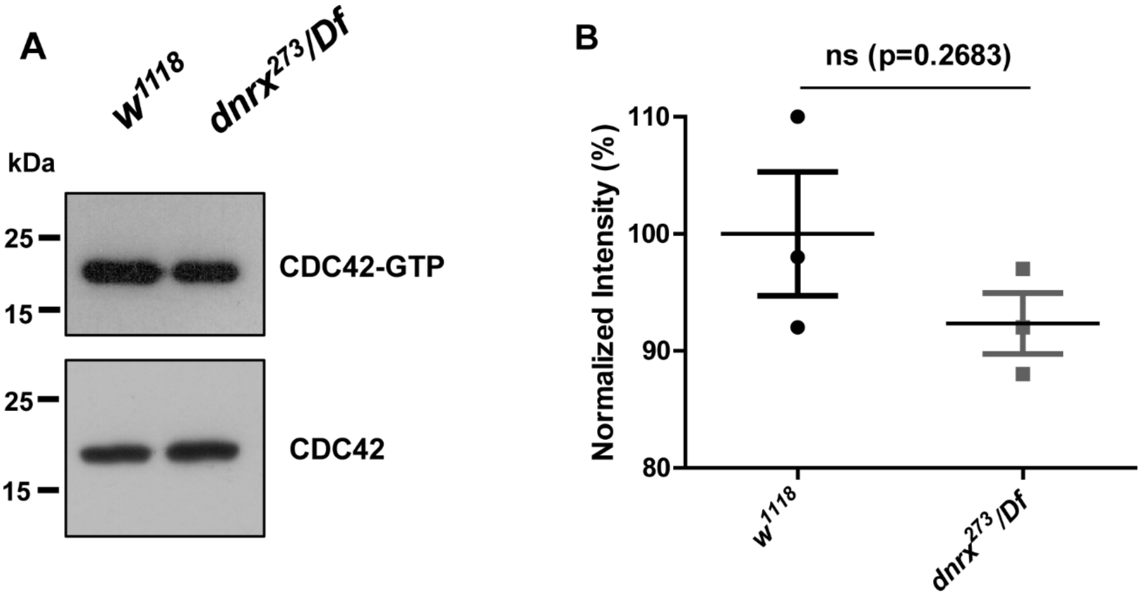

Figure S6. It has no Significant Difference of Activated Cdc42 Level between *dnrx* Mutant and *wild-type*. (A) Western blots showing the level of Cdc42-GTP and total Cdc42 in adult heads of *wild-type*, *dnrx* mutant. (B) Quantitative analysis for western blot of indicated genotypes showing that there is no significant difference of the level of activated Cdc42 in *dnrx* mutant and *wild-type*, however, there is a tendency of decrease in *dnrx* Mutant. ns: not significant. Two-tailed Student's *t* tests were used to compare genotypes.

Figure S7

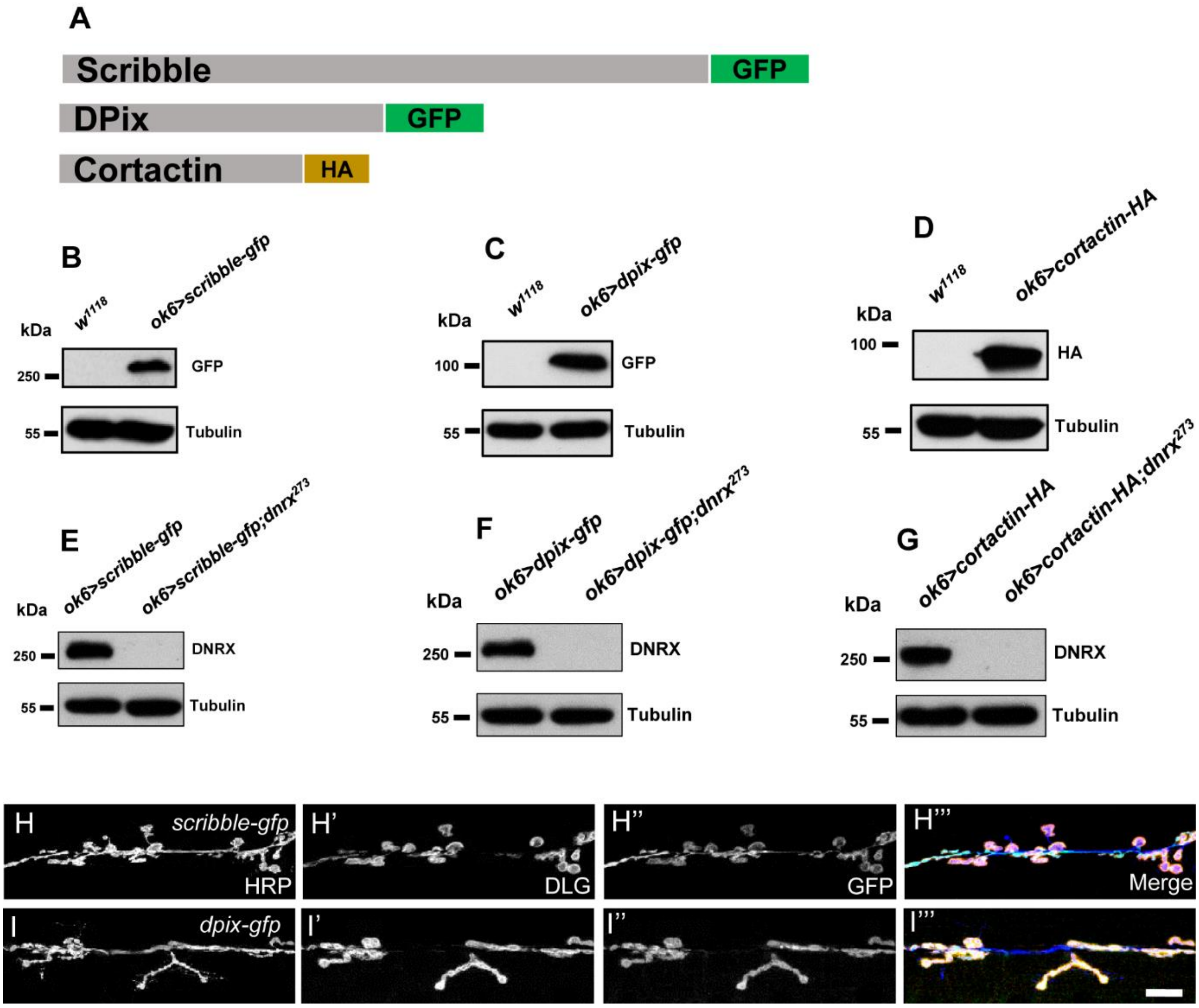

Figure S7. Identifying the Transgenic Flies and Gene Integrated Flies. (A) Schematic graph of the full-length Scribble-GFP, DPix-GFP, Cortactin-HA. (B-D) Western blot analysis of protein lysates prepared from heads of adult flies using GFP and HA antibody confirming that the protein expression of Scribble-GFP, DPix-GFP, Cortactin-HA in these transgenic flies. (E-G) Western blot analysis of protein lysates prepared from heads of adult flies using anti-DNRX antibody validating that authentication of the combined fly. (H-I''') Confocal images of third instar larvae NMJ double labeled with anti-HRP (blue), anti-DLG (red) in GFP fused Scribble and DPix transgenic flies, showing the expression of Scribble-GFP, DPix-GFP. Scale bar, 20 µm (H-I''').
